# Excitable dynamics driven by mechanical feedback in biological tissues

**DOI:** 10.1038/s42005-024-01661-2

**Published:** 2024-05-24

**Authors:** Fernanda Pérez-Verdugo, Samuel Banks, Shiladitya Banerjee

**Affiliations:** 1 Department of Physics, Carnegie Mellon University, Pittsburgh, PA, USA.; 2 Department of Physics, Yale University, New Haven, CT, USA.

## Abstract

Pulsatory activity patterns, driven by mechanochemical feedback, are prevalent in many biological systems. However, the role of cellular mechanics and geometry in the propagation of pulsatory signals remains poorly understood. Here we present a theoretical framework to elucidate the mechanical origin and regulation of pulsatile activity patterns within excitable multicellular tissues. We show that a simple mechanical feedback at the level of individual cells – activation of contractility upon stretch and subsequent inactivation upon turnover of active elements – is sufficient to explain the emergence of quiescent states, long-range wave propagation, and traveling activity pulse at the tissue-level. We find that the transition between a propagating pulse and a wave is driven by the competition between timescales associated with cellular mechanical response and geometrical disorder in the tissue. This sheds light on the fundamental role of cell packing geometry on tissue excitability and spatial propagation of activity patterns.

Multicellular systems exhibit a wide range of pulsatile and wave-like patterns during collective migration, development, and morphogenesis^1–3^. The appearance of these patterns can be attributed to various biochemical factors, depending on the specific phenomenon. These include waves of extracellular signal-related kinase (ERK)^4,5^, calcium waves^6^, periodic assembly and disassembly of myosin motors^7,8^, and the periodic release of chemoattractants^9^. Reaction–diffusion models^10–13^ and cellular automaton models^14–16^ have been widely used to study the mechanisms underlying biochemical pattern formation in multicellular systems. Mechanochemical patterns, on the other hand, have necessitated the development of new classes of models that integrate mechanical forces with chemical reactions^17–22^. For instance, the coupling of mechanical and chemical processes is particularly relevant in understanding the spatial propagation of contraction waves in *Trichoplax adhaerens*^23^, oscillatory morphodynamics in *Drosophila* amnioserosa tissue^24^, collective migration patterns^20^ and mechanical stress waves in expanding epithelial monolayers^19,25,26^. However, the role of cellular mechanics and geometry in the propagation of pulsatory signals remains poorly understood.

From the perspective of dynamical systems, pulsatory activity patterns can be regarded as manifestation of excitable dynamics, as seen in phenomena such as action potentials in nerve cells^27^ or pulses of actomyosin contractions observed in many cells^28,29^. Excitable systems share a common feedback motif: a rapid positive feedback that amplifies activity, followed by a delayed negative feedback that ultimately curtails the activity^30^. While these positive and negative feedback loops have been identified in many biological systems, how such feedback motifs can be realized in mechanical systems remains largely unexplored.

One commonly observed mechanical feedback motif in cells is stretch-induced contraction, wherein a local stretching deformation triggers the recruitment of active components that induce contraction^4,31–34^. Recent studies have utilized the concept of stretch-induced contraction to explain phenomena such as wave propagation in active elastic media^19,26^, contraction pulses in epithelial tissues^35^, cell migration patterns in vitro^20,36^, cell and tissue morphogenesis^24,37^. Specifically, all these studies focused on dynamics in active elastic media, without considering the effects of geometric disorder and viscous dissipation on mechanochemical signal propagation.

In this study, we ask how cellular viscoelasticity and packing geometry regulate the propagation of active stresses at multicellular scales. To this end, we extended the framework of the cellular vertex models^38–41^ to incorporate feedback between cell junction strain and contractility. In addition, viscous dissipation is implemented by continuous strain relaxation in cell junctions. Inspired by the mechanical stretch-triggered activation of actomyosin contractility mediated by ERK^4^, we implement a simple feedback rule in which contractility in cell junctions is activated above a threshold junctional stretch. The junction remains active for a duration commensurate with the turnover rate of active elements. Subsequently, a refractory period ensues during which junctional contractility remains inactive, potentially due to the presence of ERK inhibitors. As a result of these rules, each cell junction behaves as a mechanically excitable unit that can exist in one of three states: active, inactive, and refractory.

Our proposed model elucidates the emergence of long-range propagation of contractile pulses and different patterns of self-sustained traveling waves, such as circular, elliptic, and spiral waves. We show that these tissue-level propagation patterns are controlled by the competition between the timescales associated with active and refractory states of the junction, and the characteristic timescale of junction strain relaxation. To explain these observations, we develop an effective theory of coupled excitable junctions, capable of explaining the emergence of the quiescent, wave-like and pulse-like patterns observed in vertex model simulations. Our theoretical framework predicts that shorter junctions promote re-activation of contractility, while larger junctions facilitate the propagation of activity over a broader region of the parameter space. We validate these predictions through simulations of disordered tissues in two dimensions. We find that geometrical disorder promotes sustained wave propagation at the tissue-level, and that the ability of junctions to propagate activity increases with junction length.

## Results

### Cellular vertex model with mechanical feedback

To elucidate the emergent dynamic patterns in an excitable tissue, we use the framework of the vertex model^38–41^, where a monolayer tissue is modeled as a two-dimensional polygonal tiling. The polygons represent the cells, and the edges represent the cell-cell junctions. Each vertex i, with position $r_{i}$, is subject to friction with coefficient μ, and elastic forces and inter-cellular tensions arising from a Hamiltonian H. The Hamiltonian governing tissue mechanical energy is given by

(1) $$H=\frac{K}{2}\sum_{\alpha }{\left(A_{\alpha }-A_{0}\right)}^{2}+\sum_{\left\langle i,j\right\rangle }\Lambda l_{ij},$$

where the first energy term is a sum over all cells α, and the second term is a sum over the cell-cell junctions defined by the adjacent vertices i and j. The first term in Eq. (1) is the elastic energy that penalizes changes in cell area, where K is the bulk elastic modulus, $A_{\alpha }$ and $A_{0}$ are the actual and preferred cell areas, respectively. The second term represents an interfacial energy, with tension Λ along each cell junction of length $l_{ij}$.

Active contractile forces arise at each junction from the actomyosin cortex, generating an active force per unit length, $\Gamma_{ij}(t)$. Consequently, the active force at each vertex can be written as a length-dependent tension: $F_{i}^{\text{act}}=-\sum_{\left\langle i,j\right\rangle }\Gamma_{ij}(t)l_{ij}(\partial l_{ij}/\partial r_{i})$. Increase in active tension with edge length results in strain that is independent of the initial edge length, as observed in optogenetic experiments upon activation of cell-cell junction contractility^42^. We note that the length-dependent active tension is qualitatively similar to the perimeter-dependent contractility term in the classical vertex model energy functional^40^. However, our formulation treats each junction as a separate mechanical unit, rather than assuming homogeneous junction property across the cell.

we consider a time-dependent contractility $\Gamma_{ij}(t)$, whose dynamics depend on junctional strain and memory of mechanical state. To compute the time-evolution of each vertex, we assumed an overdamped limit, such that the equations of motion are given by:

(2) $$\mu \frac{dr_{i}}{\;dt}=-\frac{\partial H}{\partial r_{i}}+F_{i}^{act}.$$

The above equation of motion is coupled to the dynamics of junctional strain and contractility, as described below.

**Fig. 1 |:**
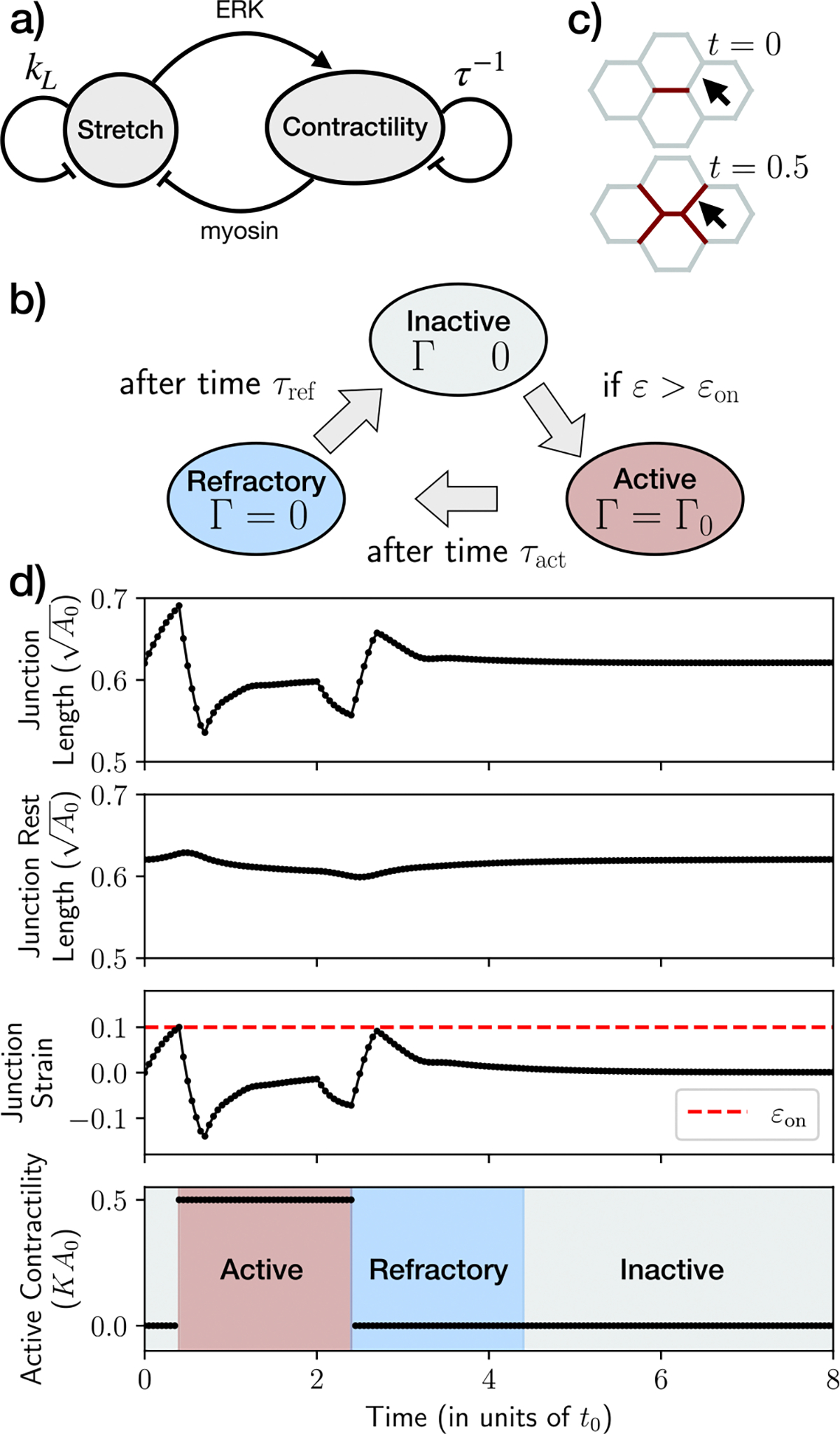
Model for stretch-induced contraction and activity dynamics in cell junctions. **a** Local junction stretch induces the activation of ERK signaling, which in turn promotes contractility. Subsequently, myosin-based contractile forces act to reduce the stretch. The triggering quantity, strain, and the resulting induced contractility are influenced by memory mechanisms, as depicted by the strain relaxation rate $k_{L}$ and the active contraction lifetime τ. **b** Junction-level rules to change its state. Red denotes active state, gray denotes inactive state, and blue denotes a refractory state. **c** Representative section of a simulated tissue with hexagonal cells, using $k_{L}=0.5$ and τ=2. Red junction color denotes active state, which spreads to neighboring junctions as they are stretched. **d** Representative dynamics of junction length, junction rest length, junction strain, and active contractility for an initially inactive junction (gray), indicated by the arrow in panel (**c**), with $\varepsilon_{\text{on}}=0.1$ (indicated by the red dashed line).

Stretch-induced contractility is a commonly observed regulatory mechanism for controlling the level of active contractile stress in cells^4,31–33,43,44^. A local stretch in cell junctions could trigger actin fiber alignment^34,45,46^, myosin recruitment^33^, and also the activation of the ERK signaling^4^ that would promote contractility. We therefore implement a simple model of cellular junctions as viscoelastic materials subject to a strain-tension feedback (Fig. 1a). Here, a local stretch triggers the activation of contractility, which in turn reduces stretch via contractile forces. Additionally, junction strain continuously relaxes over time due to viscous dissipation and contractility undergoes turnover as part of a self-regulatory mechanism.

The mechanical strain in cell junctions is defined as $\varepsilon_{ij}=(l_{ij}-l_{ij}^{0})/l_{ij}^{0}$, where $l_{ij}^{0}$ is the rest length of the junction shared by the vertices i and j. The viscoelastic nature of the junctions is modeled through rest length remodeling at a rate $k_{L}$, leading to continuous strain relaxation^42^,

(3) $$\frac{dl_{ij}^{0}}{\;dt}=-k_{L}(l_{ij}^{0}-l_{ij}).$$

Rest length remodeling is a natural consequence of actomyosin networks with turnover, where strained elements are replaced by unstrained ones^31^. The feedback between junction strain and contractility is implemented as follows. Each cell junction can exist in one of three states: inactive ($\Gamma_{ij}=0$), active ($\Gamma_{ij}=\Gamma_{0}$), and refractory ($\Gamma_{ij}=0$). While both inactive and refractory states lack contractility, refractory junctions are those that cannot be active for a duration $\tau_{\text{ref}}$, representing the timescale associated with the presence of inhibitors of contractility. The rules describing junction state changes are given below (Fig. 1b):

- Inactive junctions become active if their strain $\varepsilon_{\text{ij}}$ exceeds a threshold value $\varepsilon_{\text{on}}$.
- Active junctions become refractory after being active for a time period $\tau_{\text{act}}$.
- Refractory junctions become inactive after a duration $\tau_{\text{ref}}$.

As an example, we’ll examine the scenario depicted in Fig. 1c, d, where gray, red, and blue junctions represent the inactive, active, and refractory states, respectively. It is important to note that Fig. 1c represents a section of a larger tissue. Initially, the junction marked by the black arrow in Fig. 1c is set to an inactive state, while the middle red junction is manually activated. The contraction of the middle junction induces a moderate length increase in the inactive junction, leading to an increase in junction strain (Fig. 1d). When the strain reaches the critical value $\epsilon_{\text{on}}$, ERK signaling is activated^4^. This activation, in turn, leads to the production of ERK inhibitors^13^. The threshold value $\varepsilon_{\text{on}}$ ensures immunity to small perturbations, allowing junctions to be excitable units. ERK activation induces contractility, changing the junction state from inactive to active, increasing contractility to $\Gamma_{ij}=\Gamma_{0}$, resulting in junction contraction (Fig. 1d). The active state persists for a time period $\tau_{\text{act}}$ (red phase in Fig. 1b–d), which represents an effective timescale arising from the turnover time of actomyosin, and the inactivation of ERK. During this phase, the next inactive junction becomes activated, causing the observed length increase in Fig. 1d at $t∼0.7t_{0}$, where $t_{0}=(\mu /[\left.0.636KA_{0}\right])$. Subsequently, the initially manually activated junction returns to $\Gamma_{ij}=0$ resulting in the observed length decrease in Fig. 1d at $t∼2t_{0}$. The remaining levels of ERK inhibitors keep the junction in a refractory state, in which it can not be re-activated (blue phase in Fig. 1b–d). The initial increase in length observed in this phase is due to the sudden drop in active contractility, while the subsequent decrease in length is a result of neighbor dynamics. Finally, after a time period $\tau_{\text{ref}}$, the inhibitors reach low enough levels to take the junction back to the inactive state (final state in Fig. 1d).

Although the activation time period ($\tau_{\text{act}}$) and the refractory time period $\left(\tau_{\text{ref}}\right)$ are distinct parameters, a recent study^13^ has demonstrated that, to adequately describe the observed ERK activity waves, the characteristic timescales for ERK activation and inactivation by inhibitors tend to be similar, typically of the order of a few minutes. For simplicity, we will first focus on the case where $\tau_{\text{act}}=\tau_{\text{ref}}=\tau$, and we will refer to this timescale simply as the activation period unless otherwise specified. The strain relaxation rate $\left(k_{L}\right)$ and the activation period (τ) will jointly impact the dynamics at both the junction and tissue scales, as discussed later.

### Traveling pulse and waves in ordered tissues

To characterize the emergent dynamic states arising from junction-level mechanical feedback, we first simulated an ordered tissue, composed of 260 hexagonal cells, in a box of sides $L_{x}∼14\sqrt{A_{0}}$ and $L_{y}∼18.6\sqrt{A_{0}}$, under periodic boundary conditions. In simulations, we non-dimensionalized force scales by $K{\left(A_{0}\right)}^{3/2}$, length scales by $\sqrt{A_{0}}$, and timescales by $t_{0}=\mu /\left(0.636KA_{0}\right)$, setting $A_{0}=1$, K=1, and μ=0.636.

**Fig. 2 |:**
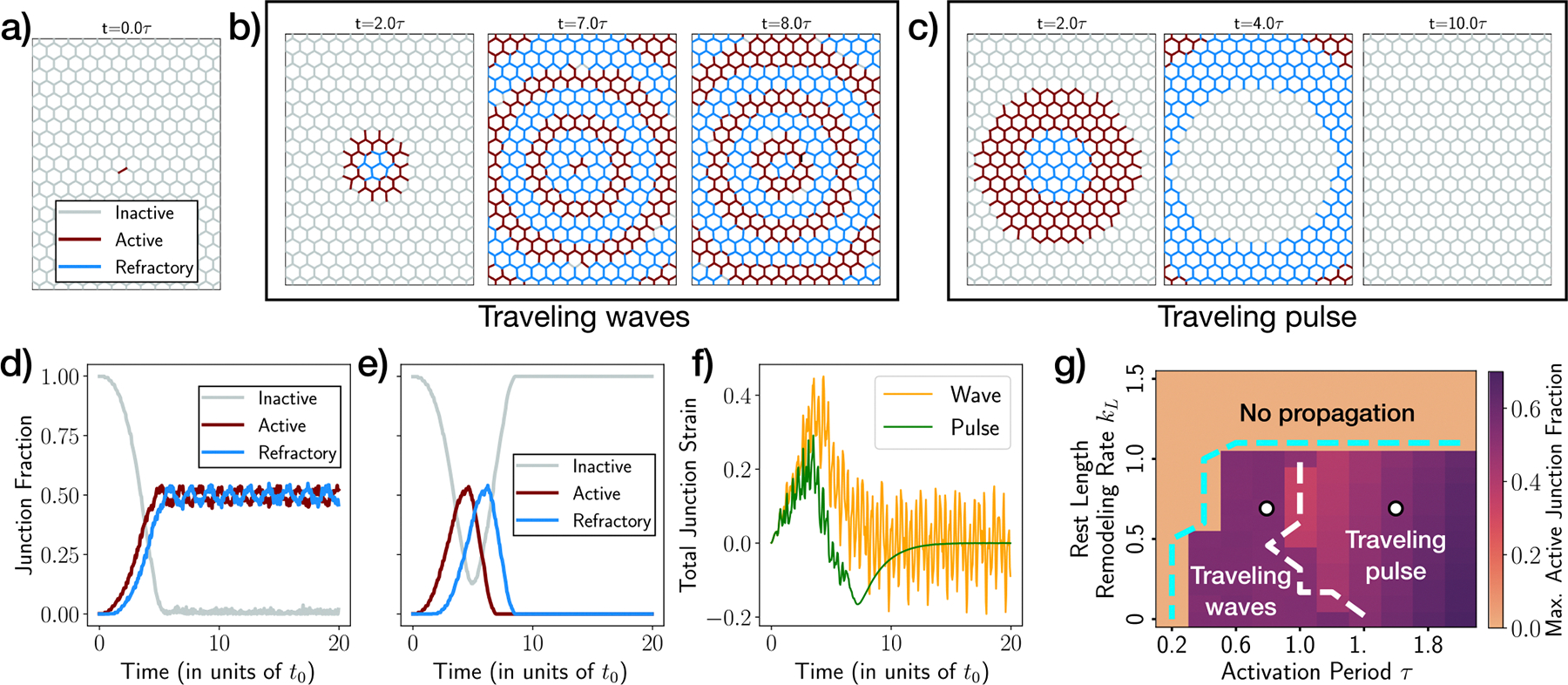
Emergent activity patterns in ordered tissues with a local junction activation. **a** Initial condition of every simulation, with a single chosen junction manually activated (red). **b** Snapshots of persistent activity waves in an ordered tissue, in a simulation using the values of $\left(\tau ,k_{L}\right)=(0.8,\;0.7)$ (left white dot in panel (**g**)). **c** Snapshots of an activity pulse traveling across an ordered tissue, in a simulation using the values of $\left(\tau ,k_{L}\right)=(1.6,\;0.7)$ (right white dot in panel (**g**)). Junction color in panels (**a–c**) denotes the state of activity: Red—active, blue—refractory, gray—inactive. **d** Fraction of junctions in inactive state (gray line), active state (red line), and refractory state (blue line), as a function of time, for activity waves shown in (**b**). Data show that at long times only active and refractory states persist, with almost no junctions in the inactive state. **e** Fraction of junctions in each state, as a function of time, for activity pulse shown in (**c**). Data show a transient pulse of activity before the entire tissue becomes inactive. Color scheme is the same as in panel (**d**). **f** Total junction strain as a function of time, for the wave (**b**) and the pulse (**c**). **g** Phase diagram showing the three distinct emergent states: traveling pulse, traveling waves, and a quiescent state with no propagation of activity. The emergent states are controlled by the rest length remodeling rate $k_{L}$ (in units of $t_{0}^{-1}$), and the activation period τ (in units of $t_{0}$). Colormap shows the value of the maximum active junction fraction. Cyan dashed line is the boundary between “No propagation” and “Traveling waves” phase, whereas white dashed line is the boundary between “Traveling waves” and “Traveling pulse” phase.

We initiate our simulations with a mechanically equilibrated tissue, where all junctions are initially in the inactive state. We then perturb the equilibrium state by manually activating a single junction positioned near the center of the simulation window (Fig. 2a). When the rate of strain relaxation is sufficiently slow, corresponding to a small value of $k_{L}$, we observe the emergence of two distinct activity patterns depending on the activation period τ: traveling waves (Fig. 2b) and traveling pulse (Fig. 2c). For small τ, we find waves of activity traveling radially outwards, as shown in Fig. 2b (Supplementary Movie 1). These self-sustaining waves are characterized by alternating rings and regions of red (indicating activity) and blue (indicating refractory) junctions. The tissue activity reaches a steady-state when the wavefront traverses the entire tissue (around $t∼5t_{0}$ in Fig. 2d). Conversely, for larger values of τ, we do not observe self-sustaining waves due to the lack of junction reactivation events. Instead, a single transient activity pulse travels across the tissue (Fig. 2c, e, Supplementary Movie 2). Over an extended time period, the tissue eventually becomes entirely inactive.

To quantify the mechanical deformation due to these traveling activity patterns, we calculated the total junction strain as $\sum_{\left\langle i,j\right\rangle }\varepsilon_{ij}$. Fig. 2f shows the dynamics of the total junction strain for both wave (Fig. 2b) and pulse-like (Fig. 2c) patterns. The pulse causes a positive peak in strain, followed by a negative peak, ultimately returning to zero strain due to mechanical relaxation. Conversely, in the traveling wave pattern, while there is a peak in strain, it eventually stabilizes as a result of activity-induced mechanical fluctuations and the relaxation of strain at the junction level.

These propagating activity states are only observed when the value of $k_{L}$ is sufficiently small. A large $k_{L}$ causes the strain in the neighboring junctions of an active junction to relax before activation can occur, resulting in a quiescent state without any propagation. To quantify the extent of tissue-scale activity, we calculated the maximum fraction of active junctions throughout the simulation. This measurement enables us to identify the phase boundary, determined by the critical value of $k_{L}$, that separates the regimes with activity propagation (either wave or pulse) from those without propagation (cyan-dashed boundary in Fig. 2g). Moreover, by quantifying the active junction fraction at the final steady state, we can differentiate between the propagating modes, leading to the delineation of the wave-to-pulse phase boundary (white-dashed boundary in Fig. 2g).

### Effective theory predicts emergent dynamic states

**Fig. 3 |:**
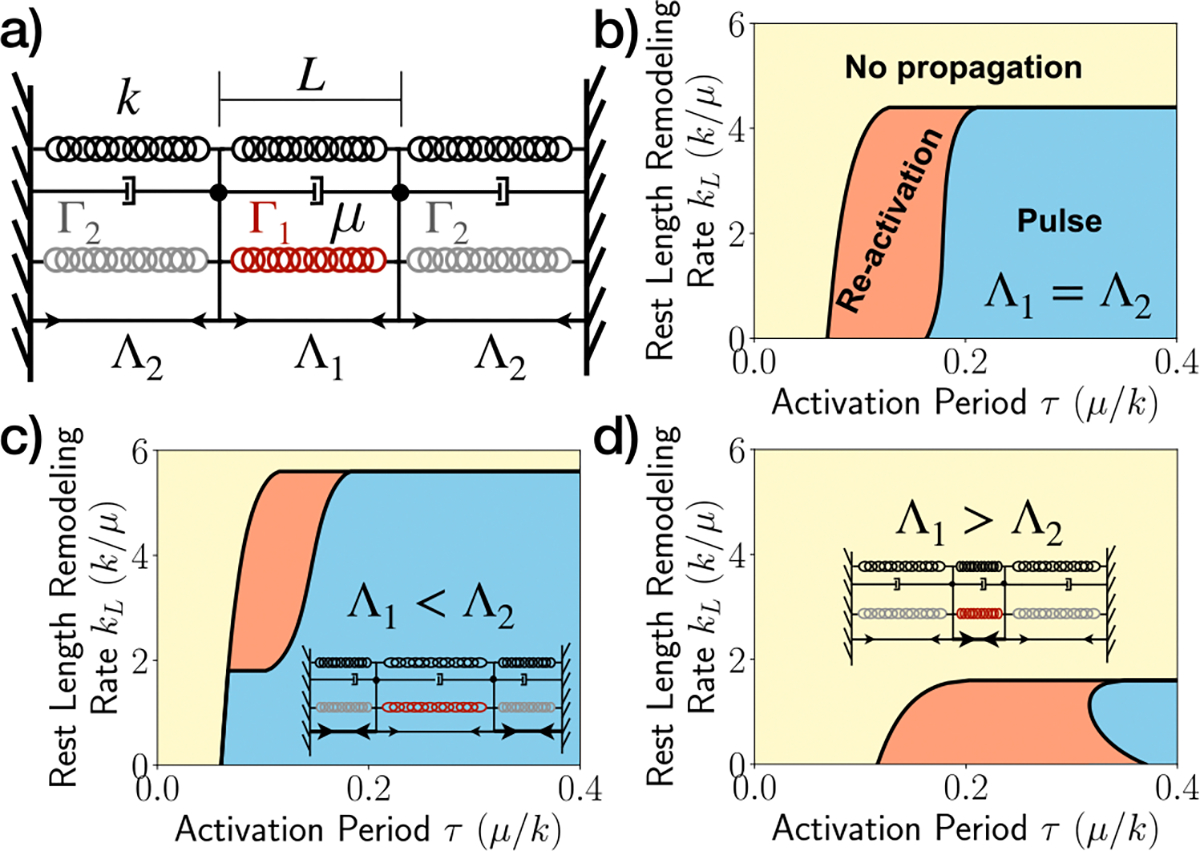
Effective model of coupled excitable junctions. **a** Schematic of a minimal three-junction model. Each junction is composed of a linear elastic element with a spring constant k and natural length L, connected in parallel with a viscous element of friction coefficient μ, and an active element with contractility $\Gamma_{1}$ (middle junction) and $\Gamma_{2}$ (end junctions). Tension in the middle junction is denoted by $\Lambda_{1}$ and those in the end junctions are given by $\Lambda_{2}$. **b** Phase diagram of the three-junction model as a function of activation period τ and rest lenght remodeling rate $k_{L}$, showing three different phases: no propagation (yellow shading), pulse propagation (blue shading) and pulse reactivation (orange shading). Here the junctions are assumed to have equal tension. **c**, **d** Phase diagrams for pulse propagation and reactivation for asymmetric junction tensions, with ($\Lambda_{1},\Lambda_{2}=0.1,\;0.2$) (c), and ($\Lambda_{1},\Lambda_{2}=0.4,\;0.1$) (**d**). All cases consider $\Gamma_{0}=1$ and $\epsilon_{\text{on}}=0.1$.

To predict the emergence of excitable pulses, quiescent states, and oscillatory patterns as functions of the strain relaxation rate $k_{L}$ and activation period τ, we developed an effective one-dimensional theory of coupled excitable junctions. Our minimal model consists of three interconnected junctions with fixed boundaries, as shown in Fig. 3a. Each unit comprises an elastic component with a spring constant k and natural length L (representing the one-dimensional version of cell elasticity), connected in parallel with a dashpot of friction coefficient μ, and an active element with contractility $\Gamma_{1,2}$. If the junction is inactive or refractory then $\Gamma_{1,2}=0$, and $\Gamma_{1,2}=\Gamma_{0}$ if the junction is active. These active and elastic elements are connected in parallel with a tensile element with line tension $\Lambda_{1,2}$. The central junction has a length $l_{1}(t)$, while the outer junctions have lengths $l_{2}(t)$. The fixed boundary conditions ensure that $l_{1}(t)+2l_{2}(t)=3L$.

The system is initialized in a mechanical equilibrium state, and we perturb it by activating the central junction ($\Gamma_{1}=\Gamma_{0},\Gamma_{2}=0$). We then let the system to evolve following the equations of motion: $\mu dl_{i}/dt=-\partial H_{eff}/\partial l_{i}$, Eq. (3), and the rules governing the junction states. The effective Hamiltonian governing the system is defined as:

(4) $$H_{eff}=\frac{k}{2}{\left(l_{1}-L\right)}^{2}+k{\left(l_{2}-L\right)}^{2}+\Lambda_{1}l_{1}+2\Lambda_{2}l_{2}+\frac{\Gamma_{1}}{2}l_{1}^{2}+\Gamma_{2}l_{2}^{2}.$$

We initially considered the scenario of symmetric junctions, wherein $\Lambda_{1}=\Lambda_{2}$. This corresponds to an ordered tissue where junction tensions and lengths are uniform. To explore the behavior of the system, we non-dimensionalized force scales by kL, length scales by L, and timescales by μ/k (setting L=1, k=1, μ=1), and numerically solved the system of equations for different values of τ and $k_{L}$, from t=0 to t=2τ. The simulation outcomes can be categorized as follows: i) If the outer junctions remain inactive throughout the simulation, it is classified as a case of *No propagation*; ii) If the outer junctions become active but the central junction does not re-activate, we observe a single *Pulse*; and iii) finally, if the outer junctions become active and the central junction re-activates, it falls into the category of *Re-activation*. Fig. 3b shows the phase diagram of the model in $\tau -k_{L}$ phase space showing the emergence of the three outcomes described above. A comparison with the phase diagram for the ordered tissue (Fig. 2g) reveals that the effective model successfully captures both key features of the vertex model: a critical value of $k_{L}$ for propagation of activity, which diminishes for small τ, and a small region of reactivation corresponding to wave-like states.

For the simple case of $k_{L}=0$, we can analytically determine the condition for propagation as $\tau >\tau_{p}$, with

(5) $$\tau_{p}=\frac{1}{2\left(3\;+\;2\Gamma_{0}\right)}\ln\left(\frac{\Gamma_{0}}{\Gamma_{0}\;-\;\left(3\;+\;2\Gamma_{0}\right)\epsilon_{on}}\right).$$

The τ-reactivation range is determined by solving an inequality of the form $Ae^{-6\tau }-Be^{-6\left(2+\Gamma_{0}\right)\tau }-\epsilon_{\text{on}}>0$, where

(6) $$A=\frac{2\Gamma_{0}}{3\;+\;\Gamma_{0}}\left(1-e^{-2\left(3+\Gamma_{0}\right)\tau_{p}}\right)e^{6\tau_{p}},$$

(7) $$B=\frac{2\Gamma_{0}}{3\;+\;2\Gamma_{0}}\left(1-e^{-2\left(3+2\Gamma_{0}\right)\tau_{p}}\right)e^{2\left(3+2\Gamma_{0}\right)\tau_{p}}.$$

The above inequality cannot be analytically solved in general, but we can solve several limiting cases to gain intuition on the dynamics. Initially, we examine the case where $\epsilon_{\text{on}}=0$. Here, $\tau_{p}=0$, indicating that activity propagates regardless of the activation period t. Consequently, A=B=0. Thus, in the absence of rest length remodeling and critical strain threshold, only one outcome prevails: propagation of activity pulse. Subsequently, when considering $\Gamma_{0}=1$, as in the numeric solution shown in Fig. 3b, the left-hand side of the inequality assumes a cubic form for y=exp(−6τ). Within the range $0.077<\epsilon_{\text{on}}<0.2$, the inequality yields a range of activation timescales $\tau_{1}\left(\epsilon_{\text{on}}\right)<\tau <\tau_{2}\left(\epsilon_{\text{on}}\right)$ where reactivation occurs. The exact positioning of $\tau_{1}$ relative to $\tau_{p}$ varies depending on $\epsilon_{on}$. Specifically for $\epsilon_{on}=0.1$ (as considered in Fig. 3b), we find that $\tau_{p}<\tau_{1}<\tau_{2}$, with $\tau_{1}-\tau_{p}=0.003$. It is noteworthy that across all the plots shown in Fig. 3, $\tau_{1}-\tau_{p}\leq 0.025$, thus the contours of $\tau_{1}$ are not displayed.

We then used the one-dimensional effective model (Fig. 3a) to investigate the role of disorder in the propagation of activity. Disorder was introduced by removing the condition of homogeneous line tension, letting $\Lambda_{1}\neq \Lambda_{2}$. First, we analyzed the case $\Lambda_{1}<\Lambda_{2}$. Due to identical mechanical properties of each junction before activation (other than tension values), the initial equilibrium state featured a central long junction $\left(l_{1}>L\right)$ flanked by two shorter junctions $\left(l_{2}<L\right)$ (Fig. 3c). By solving the system of equations numerically, we found that the larger junction $\left(l_{1}>L\right)$ could propagate activity over a broader region in the ($\tau ,\;k_{L}$) parameter space, while the re-activation region is substantially diminished. This is because larger junctions produce greater active contractile forces, while shorter neighboring junctions require a lower extension to achieve the strain threshold for activation $\varepsilon_{\text{on}}$. Conversely, when $\Lambda_{1}>\Lambda_{2}$, the opposite behavior was observed (Fig. 3d). Our effective model thus reveals two main effects of the geometrical heterogeneity (or disorder) on cellular response to active contractility. Large junctions promote propagation of activity, while shorter junctions facilitated re-activation, leading to oscillatory patterns.

### Tissue disorder promotes self-sustained wave propagation

**Fig. 4 |:**
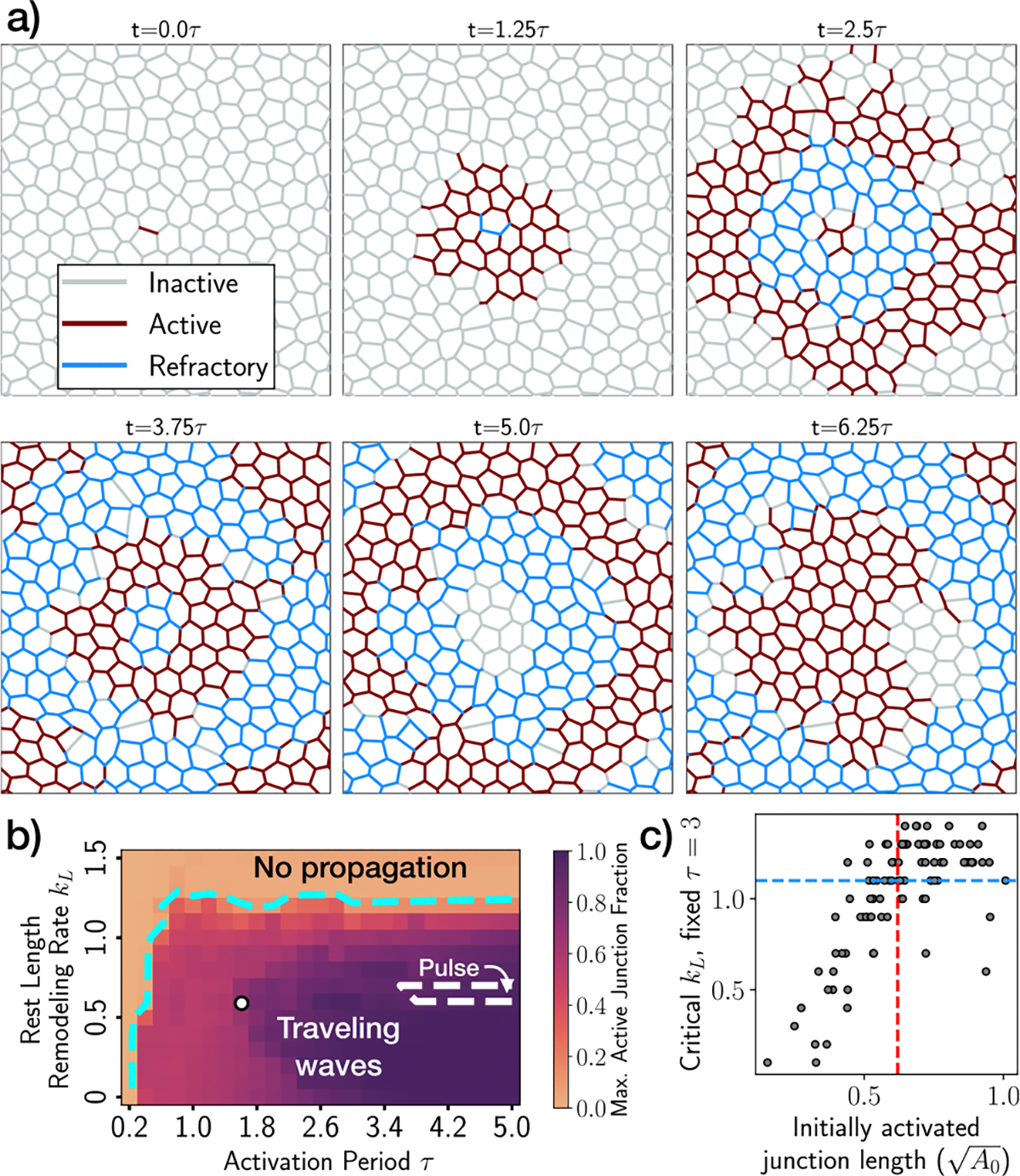
Sustained propagation of activity waves in disordered tissues. **a** Snapshots of activity waves traveling across a disordered tissue, using $\left(\tau ,k_{L}\right)=(1.6,\;0.7)$ (white dot in (**b**)). Colored segments represent inactive (gray), active (red), and refractory (blue) junctions. (**b**) Phase diagram for the emergence of waves, pulses and quiescent states, varying strain relaxation rate $k_{L}$ (in units of $t_{0}^{-1}$) and activation period τ (in units of $t_{0}$). The cyan boundary indicates a maximum active junction fraction equal to 0.1, whereas the white dashed line is the boundary between 'Traveling waves' and 'Traveling pulse'. **c** Scatter plot of the critical rest length remodeling rate $k_{L}$ for a fixed τ=3, as a function of the initially activated junction length, for 100 different chosen junctions. The critical $k_{L}$ follows the same definition as the cyan boundary in (**b**). Red-vertical line represents the junction length in the ordered hexagonal tissue. Blue-horizontal line represents the critical $k_{L}$ in the ordered tissue.

Motivated by the predictions of the effective model on the impact of geometric heterogeneity, we now investigate the effect of disorder in cell packing geometry on activity propagation in two-dimensional tissue simulations. To this end, we constructed a tissue comprising 208 cells, within a rectangular box with dimensions approximately equal to $L_{x}∼14\sqrt{A_{0}}$ and $L_{y}∼15\sqrt{A_{0}}$, subject to periodic boundary conditions. In these simulations, all mechanical properties at cell and junction levels are the same, with disorder restricted to geometric heterogeneity only. The initial state of the tissue corresponded to a state of mechanical equilibrium, characterized by varying junction lengths and polygon sidedness, as depicted in Fig. 4a.

As previously, we activated a randomly chosen cell junction (see Fig. 4a, t=0.0τ), and let the tissue evolve from t=0 to t=20, for different values of strain relaxation rate $k_{L}\in (0,\;1.5)$ and activation period τ∈(0.2,5.0). By measuring the maximum active junction fraction (Fig. 4b), we again observe that propagation occurs below a critical $k_{L}$, for sufficiently large τ. Unlike in ordered tissues (Fig. 2g), wave states are now possible for a wide range of τ values, and propagating solitary pulse only occurs in particular cases with exceedingly large activation periods. Consistent with the predictions of the one-dimensional effective model, we find that the presence of short junctions in disordered tissues promotes junction reactivation, thereby facilitating the emergence of self-sustaining wave-like states. As an illustrative example, Fig. 4a (corresponding to the white dot in Fig. 4b) represents a wave-like state arising in a tissue with parameters ($\tau =1.6,k_{L}=0.7$) (Supplementary Movie 3), which led to pulse propagation in the ordered tissue (Fig. 2g). Interestingly, the junction that is reactivated by the end of an oscillatory cycle need not necessarily be the same one initially chosen for activation. This introduces a non-local effect of disorder in promoting sustained wave-like patterns. We find that the critical $k_{L}$ required for wave propagation increases with the length of the initially activated junction (Fig. 4c), as predicted by the one-dimensional effective model.

Interestingly, we observe that certain long junctions, surrounded by shorter ones, retain their inactive state throughout the entirety of the simulations. This phenomenon is rooted in the longer activation period necessary to propagate activity in such configurations, as predicted by the one-dimensional effective model (Fig. 3d). In certain scenarios, this effect merely delays the onset of activation, whereas in others, it renders activation unattainable.

### Controlling the geometry of wavefronts

Our theory and simulations have elucidated that the propagation of activity at the tissue scale is governed by two distinct characteristic timescales of the system: the activation period (τ) (taken to be equal to the refractory time) and the rest length remodeling timescale $\left(k_{L}^{-1}\right)$. We now investigate the impact of varying the activation ($\tau_{\text{act}}$) and refractory periods ($\tau_{\text{ref}}$) on the resulting dynamic patterns that emerge within the tissue. In particular, we show that the ratio of activation to refractory period controls the geometry of wave patterns.

**Fig. 5 |:**
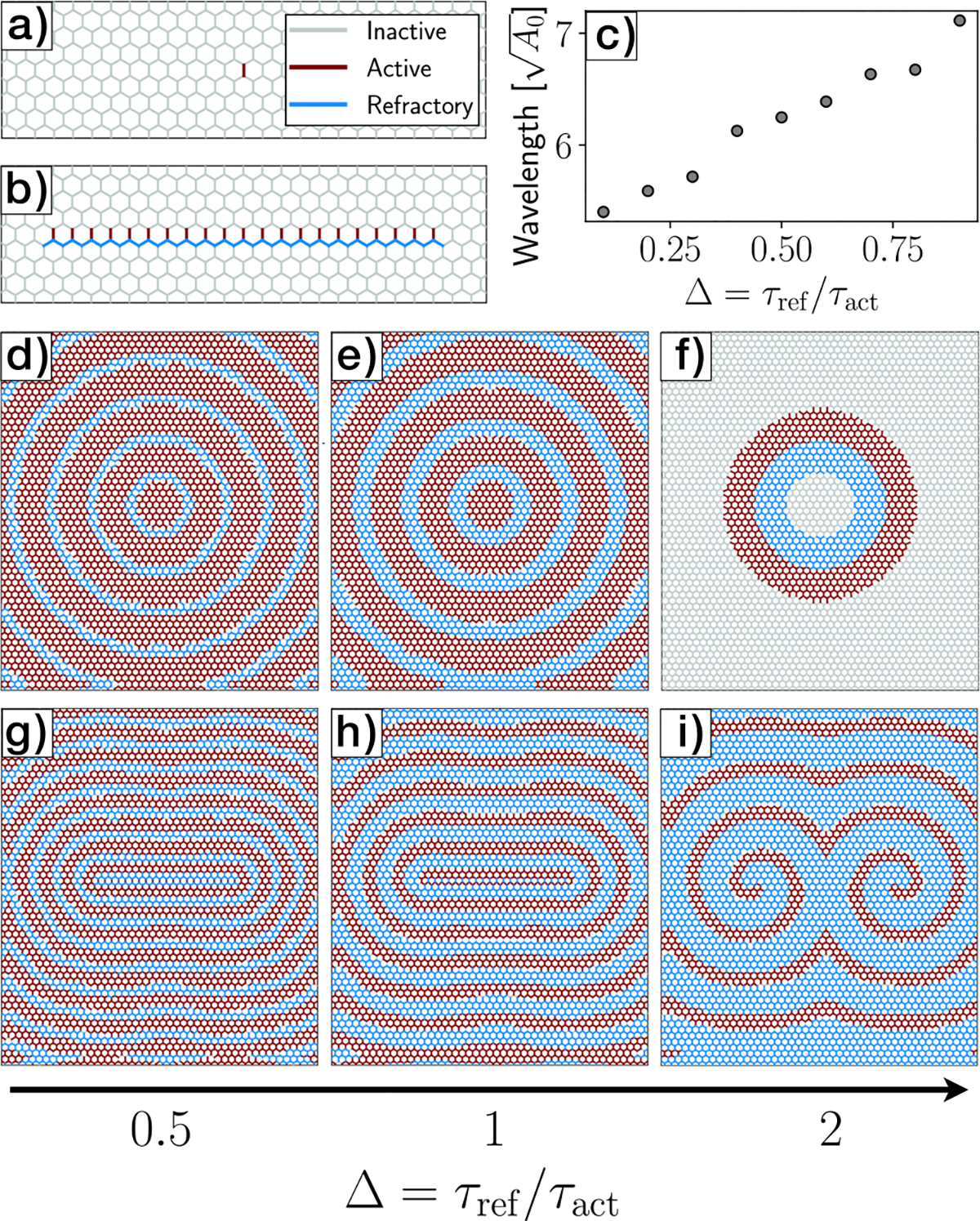
Emergent dynamical patterns arising in an ordered tissue. The tissue is composed of 2759 cells, varying $\Delta =\tau_{\text{ref}}/\tau_{\text{act}}$. Colored segments represent inactive (gray), active (red), and refractory junctions. **a, b** Zoomed in configuration of two different initial conditions for junction states: **a** single junction activation, and **b** partial row activation with neighbors underneath in refractory states. **c** Wavelength versus Δ, for the initial condition shown in (**a**). **d–f** Collective dynamics arising from the initial condition in (**a**), for different values of $\tau_{\text{ref}}$, with $\tau_{\text{act}}=\;2$. **g–i** Collective dynamical states arising from the initial condition (**b**), for different values of $\tau_{\text{ref}}$, with $\tau_{\text{act}}=1$. Panels **d, e, g–i** represent self-sustained waves, while panel **f** represents a solitary traveling pulse. All these simulations consider $k_{L}=0.5$, and integration time step Δt=0.05.

We initiated the simulations by activating a single junction (Fig. 5a) or a partial row of junctions (Fig. 5b) within the tissue, while the remaining junctions remained in the inactive state. We find that the ratio of the refractory period to the activation period, $\Delta =\tau_{\text{ref}}/\tau_{\text{act}}$, controls the wavelength of the propagating waves (see Figs. 5c–e). Specifically, at higher values of Δ, propagating waves fail to materialize, and instead, we observe the presence of a solitary traveling pulse of activity (Fig. 5f).

Inspired by self-sustained spiral patterns observed in excitable systems^16,47–49^, we inquired whether we could design an initial state that would break the circular symmetry of the emergent wavefronts. Previous theoretical work using a three-state (inactive, active, refractory) cellular automata model has shown that spiral waves can emerge from an initial state consisting of a layer of excited cells and an adjacent layer of refractory cells^50–52^. We therefore initialized our simulations by activating a partial row of junctions, followed by neighbors underneath in refractory states, while the remaining junctions remained in inactive states (Fig. 5b). This initial condition leads to elliptical wavefronts for the cases of Δ<1 (Fig. 5g) and Δ=1 (Fig. 5h, Supplementary Movie 4). Similar to the case of single junction activation, smaller values for Δ decrease the wavelength of the traveling wavefront (Fig. 5g). For higher values of Δ we observe the emergence of a pair of self-sustaining spirals (Fig. 5i, Supplementary Movie 5). This can be explained as follows. The initial condition of a partial row of active junctions followed by a layer of refractory junctions instigates two distinct patterns. Firstly, it leads to the emergence of a propagating wavefront. Secondly, it initiates the formation of two open ends within the wavefront, resulting initially in the addition of an excited element and subsequently in the development of a curved wave segment. This curved segment then propagates outward, adopting a spiral shape. Thus, each open end leads to the emergence of an spiral. Note that due to the periodic boundary conditions in our model, the least number of open ends that can be created is two. These results show that by designing appropriate initial states and disparate timescales for junction activation and refractory periods, the geometry and wavelength of the emergent wavefronts can be precisely controlled in our model.

## Discussion

Excitable dynamics are widespread in nature, involved in diverse biological and chemical processes, including action potentials in nerve cells^27^, calcium wave propagation^53^, the Belousov-Zhabotinsky chemical reaction^54^, pulsatory Rho dynamics^28,55^ and Ras-GTP (guanosine triphosphate)^56^ signaling networks. Although excitable dynamics have been well-studied in the context of biochemical networks, there are a few examples of mechanical circuits that display excitable behavior^57^. In this work, we have elucidated the minimal design principles for mechanical excitability in living tissues using the framework of the cellular vertex model. Our extended vertex model incorporates agent-based rules at the level of cell junctions, representing coarse-grained biochemical reactions that connect junction deformations with the activation of contractility. Through this model, we have demonstrated how the timescales regulating excitability and cell-level geometrical disorder control the formation of different spatio-temporal patterns and facilitate long-range signal propagation in the tissue.

Prior work has examined pattern formation in excitable tissues, considering various triggering factors, including cell-level tension^35^, cell size^36^, and active ERK concentration^4^. However, none of these models have considered the effects of viscous dissipation or explored the potential roles of geometrical disorder in pattern formation dynamics. Here we investigated the role of viscous dissipation and geometrical disorder on tissue-level pattern formation. We showed that mechanical strain can trigger the emergence of three tissue-level states: quiescent (no propagation), traveling waves, and traveling pulses. These states arise from the interplay between the characteristic timescales associated with junction activation, inactivation, and refractory states.

To explain these emergent dynamics, we have developed an effective junction-scale theory that qualitatively captures the observed behaviors in the vertex model. Our model also provides insights into the impact of geometrical tissue disorder on tissue-level activity states, demonstrating that large junctions promote propagation, while small junctions facilitate re-activation. These predictions have been corroborated through two-dimensional vertex-like simulations, although experimental validation in epithelial tissues remains an avenue for future exploration.

Furthermore, we have demonstrated that the geometry of the emerging traveling wavefronts is influenced by the initial state of junctions and the ratio between the durations of junction active states and refractory states. This intricate interplay results in variations in wavelengths, transitions from waves to pulses, formation of elliptic wavefronts, and pairs of self-sustained spiral wavefronts. The predicted patterns arising from specific initial junction states could potentially be experimentally tested using optogenetic tools to spatially activate myosin contractility^58^, ERK^59^, and Fluorescence resonance energy transfer (FRET) imaging to visualize the resulting patterns^4^.

## Methods

The custom simulation code for the vertex model, implemented using Python 3, uses the Euler integration method to integrate the differential equations. All the simulation codes can be accessed on GitHub (https://github.com/BanerjeeLab/Excitable_Tissue). In implementing T1 transitions, a similar approach to that described in a previous work by some of us was adopted^60^. This involves enforcing the creation and instantaneous resolution of a 4-fold vertex whenever a junction’s length becomes smaller than $l_{T1}$. A newly created junction is set to have $l=l^{0}=1.5l_{T1}$ and be in the inactive state. The simulations encompassed tissues of varying sizes, as specified in the respective figure captions. Default model parameters used in the simulations are listed in Table 1. The numerical analysis of the one-dimensional effective model was done in Mathematica 12.

## Supplementary Material

Supplementary Movie 1

Supplementary Movie 2

Supplementary Movie 3

Supplementary Movie 5

Supplementary Movie 4

## Figures and Tables

**Table 1 |:** Default model parameters

Parameter	Symbol	Value	Units
Area elastic modulus	*K*	1	*K*
Preferred area	*A*_0_	i	*A*_0_
Friction coefficient	*μ*	0.636	*t*_0_ *KA*_0_
Line tension	Λ	0.1	$KA_{0}^{3/2}$
Active contractility	Γ_0_	0.5	*KA*_0_
Strain relaxation rate	*k*_L_	1	${t_{0}}^{-1}$
Activation strain	ε_on_	0.1	-
Length threshold for T1 event	$I_{T_{1}}$	0.01	$\sqrt{A_{0}}$
Integration time step	Δ*t*	0.01	*t*_0_

## Data Availability

The data that support the findings of this study can be generated by running the codes available on GitHub.
